# Pediatric Hepatobiliary Lithiasis in Homozygous Sickle Cell Disease: Laboratory Insights Into Cholestasis and Hemolysis

**DOI:** 10.7759/cureus.102913

**Published:** 2026-02-03

**Authors:** Faralahy H Rakotonjafiniarivo, Tahianasoa Randriamampianina, Stephania Niry Manantsoa, Miora Koloina Ranaivosoa, Aimée O Rakoto Alson

**Affiliations:** 1 Medical Biochemistry, University of Antananarivo, Antananarivo, MDG; 2 Hematology, University of Antananarivo, Antananarivo, MDG

**Keywords:** cholestasis, hemolysis, microlithiasis, pediatric, sickle cell disease

## Abstract

Chronic hemolysis in sickle cell disease (SCD) predisposes patients to hepatobiliary complications, including pigment gallstones. We report a 13-year-old girl with hemoglobin SS SCD who presented with right upper quadrant pain, progressive jaundice, and fever. Laboratory evaluation revealed severe anemia with reticulocytosis, mixed hyperbilirubinemia, elevated lactate dehydrogenase, normal cholestatic enzymes, and marked C-reactive protein elevation. Imaging confirmed microlithiasis without significant bile duct obstruction. Supportive care included transfusion, hydration, antibiotics, and pain control. Elective cholecystectomy was planned after stabilization. This case underscores the diagnostic value of combined hemolysis and cholestasis markers for early recognition of hepatobiliary complications in pediatric patients with SCD.

## Introduction

Sickle cell disease (SCD) is an inherited hemoglobinopathy characterized by chronic hemolysis, which leads to multiple systemic complications, with hepatobiliary involvement being a frequent cause of morbidity [1]. Persistent hemolysis results in increased production of unconjugated bilirubin, promoting the formation of pigment gallstones, sometimes from early childhood [2]. Biologically, hepatobiliary complications in SCD manifest as complex laboratory abnormalities [3], which can make interpretation of liver function tests challenging. Early identification of these biochemical disturbances is essential to guide diagnosis and management. Here, we report a case of a pediatric patient with SCD presenting with a hepatobiliary lithiasic complication, highlighting the value of a systematic biologically driven approach in the evaluation of abdominal pain and jaundice in patients with SCD.

## Case presentation

This case report was conducted in accordance with the ethical principles of the Declaration of Helsinki. Ethical approval was obtained from the local ethics committee of Société Malgache de Biologie Clinique (SOMABIO). Written informed consent was obtained from the patient’s parent/legal guardian for publication of this case report and any accompanying anonymized clinical data.

A 13-year-old female patient with homozygous (HbSS) SCD, diagnosed at the age of five, with a history of vaso-occlusive crises and irregular follow-up, was admitted to the Hematology Department of Joseph Ravoahangy Andrianavalona Hospital, Antananarivo, Madagascar. She presented with severe right upper quadrant abdominal pain associated with progressive jaundice and intermittent fever for three days.

On clinical examination, the patient was alert but asthenic, with a moderate fever of 38.5°C and diffuse cutaneous-scleral jaundice. Abdominal palpation revealed moderate tenderness in the right hypochondrium with mild hepatomegaly, without guarding or rigidity. The spleen was palpable approximately 2 cm below the left costal margin. No cutaneous or mucosal hemorrhagic signs were observed. Laboratory findings are summarized in Table 1.

**Table 1:** Laboratory profile of hemolysis and cholestasis in a pediatric sickle cell patient. WBC: white blood cells; LDH: lactate dehydrogenase; ALP: alkaline phosphatase; AST: aspartate aminotransferase; ALT: alanine aminotransferase; CRP: C-reactive protein; GGT: gamma-glutamyl transferase.

Parameters	Patient value	Normal range	Unit	Interpretation
Hemoglobin	4.7	12 - 16	g/dL	Severe anemia
WBC	8.44	4 - 10	Giga/L	Normal
Platelets	56	150 - 450	Giga/L	Severe thrombopenia
Reticulocytes	5	0.5 - 2.5	%	Marked reticulocytosis
LDH	660	180 - 420	U/L	Elevated
Total bilirubin	498.80	<20	µmol/L	Major hyperbilirubinemia
Conjugated bilirubin	249.40	<9	µmol/L	Partial cholestasis
ALP	99	38 - 126	UI/L	Normal
GGT	44	9 - 36	UI/L	Mild elevation
AST	54	11 - 34	UI/L	Mild elevation
ALT	21	<34	UI/L	Normal
CRP	428.1	<6	mg/L	Severe inflammation

Complete blood count (CBC) showed severe normocytic anemia with hemoglobin of 4.7 g/dL and marked reticulocytosis. White blood cell count was within the normal range, while platelet count revealed severe thrombocytopenia at 56 Giga/L. Hemolysis markers indicated elevated lactate dehydrogenase (LDH) at 660 U/L, associated with mixed hyperbilirubinemia (total bilirubin = 498.8 µmol/L), with conjugated bilirubin accounting for approximately 50% of the total bilirubin. Serum total and conjugated bilirubin were measured using an automated wet chemistry method, and indirect bilirubin was calculated. Liver function tests showed normal alkaline phosphatase (ALP), gamma-glutamyl transferase (GGT), and alanine aminotransferase (ALT), while aspartate aminotransferase (AST) was slightly elevated at 54 U/L. C-reactive protein (CRP) was markedly elevated at 428.1 mg/L. Viral hepatitis serologies (A, B, and C) were negative.

**Figure 1:**
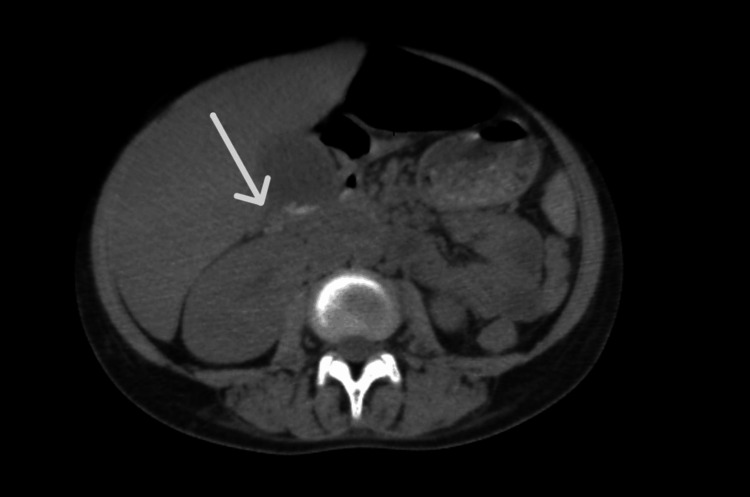
Contrast-enhanced abdominal CT scan demonstrating intrahepatic bile duct dilatation. Contrast-enhanced abdominal CT scan (axial view) showing marked dilatation of intrahepatic bile ducts (white arrow), consistent with cholestasis.

Abdominal ultrasound revealed homogeneous hepatomegaly with a cluster of gallstones, without signs of acute complications. Abdominal CT confirmed the presence of dependent microlithiasis, consistent with sickle cell hepatopathy (Figure 1).

The patient was hospitalized for supportive management, including blood transfusion, broad-spectrum antibiotics, intravenous hydration, pain control, and correction of associated biochemical abnormalities. Elective cholecystectomy was planned after clinical and hematologic stabilization. Measures for vaso-occlusive crisis prevention and regular hematology follow-up were recommended.

## Discussion

Hepatobiliary complications are a common cause of morbidity in patients with SCD [1]. Gallstone formation, particularly pigment stones, is closely linked to chronic hemolysis or altered erythropoiesis and may occur early in these patients [1,4]. This case illustrates the characteristic laboratory profile of a lithiasic complication in a patient with homozygous SCD. Severe normocytic anemia with marked reticulocytosis reflects chronic hemolysis, the central pathophysiological mechanism of SCD. Accelerated red blood cell destruction leads to excessive production of unconjugated bilirubin, promoting pigment gallstone formation in the gallbladder [1,5]. This mechanism explains the high prevalence of gallstones in patients with SCD, often from a young age.

In this case, hyperbilirubinemia was mixed, with elevated unconjugated bilirubin from chronic hemolysis and a significant increase in conjugated bilirubin, indicating cholestasis secondary to biliary obstruction [2,6]. Despite moderate imaging signs of cholestasis, ALP and GGT remained within normal ranges. This can be explained by the fact that, in certain mild or partial hepatobiliary involvement, particularly when there is no significant bile duct dilatation or major obstruction, cholestatic enzymes do not rise enough to exceed diagnostic thresholds for cholestasis [7]. Furthermore, in children, physiological variations and the predominance of bilirubin elevation due to chronic hemolysis can mask subtle increases in ALP or GGT [8]. Moderate AST elevation suggests secondary hepatocellular involvement without severe cytolysis, although it could also result from red blood cell lysis, as RBCs contain AST but not ALT [9]. LDH elevation serves as an indirect yet relevant biomarker of chronic intravascular hemolysis [10]. Combined with reticulocytosis and indirect hyperbilirubinemia, it helps differentiate hepatobiliary involvement secondary to hemolysis from primary liver disease [11]. In this case, the Tokyo Guidelines were referenced to aid in excluding acute cholangitis [12]. The absence of marked leukocytosis and thrombocytosis, typically observed in acute cholangitis, along with only moderate CRP elevation, suggests a nonspecific inflammatory process. Excluding viral etiology through negative hepatitis A, B, and C serologies is essential for the biological assessment, particularly in sickle cell patients frequently exposed to transfusions [13]. This supports the hypothesis that the observed laboratory abnormalities are primarily linked to the lithiasic complication and underlying sickle cell hepatopathy [1]. The patient’s severe thrombocytopenia (56 G/L) is likely due to functional hypersplenism [14]. Moderate splenomegaly, as indicated by the palpable spleen, can lead to platelet sequestration and destruction, a common finding in pediatric sickle cell patients.

In selected pediatric sickle cell patients with gallstones, elective laparoscopic cholecystectomy is frequently recommended as a standard treatment to prevent future complications, even in the absence of severe symptoms [15]. Hydroxyurea is effective as a pharmacological treatment in reducing painful vaso-occlusive crises and the number of hospitalizations [16].

## Conclusions

This case highlights the importance of systematic laboratory evaluation and regular follow-up in sickle cell patients presenting with abdominal pain and jaundice. The combination of chronic hemolysis markers and a biochemical cholestasis pattern should alert clinicians to the possibility of hepatobiliary complications and prompt early imaging. In resource-limited settings, careful interpretation of laboratory parameters remains a fundamental tool for guiding diagnosis and optimizing patient management.
